# The Use of the Red Cross

**Published:** 1914-10-17

**Authors:** J. B. Cornish

**Affiliations:** Hon. Sec. West Cornwall Dispensary and Infirmary, Penzance.


### THE EDITOR'S LETTER-BOX.

### The Use of the Red Cross.

*To the Editor of* The Hospital.

Sir,—I have read with interest your Note on this subject in your issue of the 10th. Cambridge Town Council are not alone in their experience. The Home Office circular of September 22, which I believe has been sent to all police authorities, suggests that it may be desirable to prosecute. The penalty is £10.

On seeing this circular I wrote to the Army Council asking for permission to fly the Red Cross flag on our hospital and at our convalescent home. I received a reply that "the terms of the Geneva Convention do not permit of the Red Cross flag being used except by medical units or establishments of an army."

I then asked what flag civil hospitals were entitled to use, and have the answer that "only hospitals which have been taken over by the military authorities are entitled to fly the Red Cross flag. Civil hospitals not so taken over are not allowed this privilege."

This, of course, did not answer my question, and the difficulty seems to be a technicality which could be got over if there was any disposition to do so. But as the position now is, the civil hospitals have no flag or badge which they can use.

As the matter is not merely a question of sentiment or decoration, I hope that those interested in our larger and more influential hospitals will take it in hand at once, and if the War Office cannot be persuaded to change their decision, arrange for the adoption by civil hospitals of a new mark, such as the same cross in red on a yellow ground, or a similar cross in blue on a white ground.—Yours truly, J. B. Cornish, Hon. Sec.

West Cornwall Dispensary and Infirmary, Penzance.

[Our Note last week was specially in reference to the use of the Red Cross on annual reports, and we should be glad to have further correspondence on the subject. In regard to the flag a hospital may fly, the whole question was gone into expertly in The Hospital of August 2, 1913, p. 2 of the *Institutional Worker* Section. Detailed treatment was also given to the subject in The Hospital of June 17, 1911, p. 293, and in our issue for June 10, 1911, p. 263.—Ed. The Hospital.]

